# Housekeepers’ Department

**Published:** 1869-04

**Authors:** 


### Housekeepers' Department.

BY A HOUSEKEEPER.

Containing useful hints, and simple, well tried receipts, especially adapted for the use of young house-keepers with limited incomes.

Care has been taken to make the receipts plain to persons ignorant of cookery, so that a lady may prepare any of the dishes without assistance. Cooking for the sick will also receive attention. A good nurse is frequently as desirable as a skillful physician, and food for the sick is often of the first importance.—We have carefully gathered together many strengthening prescriptions which we hope may be of use to our invalid readers.

---

**Cocoanut Pie.**

One cocoanut grated, one quart of milk, four eggs, sweeten.

---

**Eggs and Wine.**

Take a fresh egg, beat it until it becomes thick, sweeten it to your taste and add a tablespoonful of wine.

---

**Fried Cakes.**

One-half cup of butter, one of sugar, two eggs, a cup of milk, one-half teaspoonful of soda, one of cream of tatar. Mix, and roll, not too thin; fry in boiling lard.

---

**A Cooling Lemon Drink.**

Pare two lemons, be careful to take off all the white skin, cut them in thin slices and pour over them one pint of boiling water. When cold, strain and sweeten with loaf sugar, and if found too sour add cold water.

---

**Egg and Milk.**

Beat separately the yolk and white of a fresh egg; add to the yolk a glass of good milk, sweeten it to your taste, then stir in the white. Fresh eggs are well known to contain strengthening qualities and we especially recommend these drinks to the weak.

---

**Arrowroot.**

Take care to get the *very best* arrowroot. Mix a dessert spoonful with a little cold water till quite smooth. Boil half a pint of milk, pour it on the arrowroot while boiling, stirring it all the time. Add a lump or two of sugar and a little lemon peel.

---

**Beef Tea.**

Cut two pounds of lean beef into small pieces put it into a jar without water, cover closely, place the jar in the oven for three or four hours until every drop of the gravy is out of the meat, then mix with boiling water to the strength required.

**Wine Jelly.**

Clarify half a pound of loaf sugar, dissolve one ounce of isinglass in a small quantity of water, strain it into the syrup; when nearly cold add half a pint of wine, mix it well and pour it into a bowl. For the convalescent this jelly is very nutritious.

### FOR WELL FOLKS.

**Cottage Pudding.**

One egg, one-half cup of sugar, two cups of milk, two tea-spoonsful of cream of tartar, one of soda, two and one-half table-spoonsful of melted butter. Beat all together quickly and bake in a three pint basin. This also makes a good plain jelly cake.

**Tapioca Cream.**

Soak two table-spoonsful of tapioca in a teacup of water over night; add to this a pint of milk, and place in boiling water to heat. Beat the yolks of two eggs and three-fourths of a cup of sugar together and add to the milk, stirring it quickly until it forms a custard. Remove from the fire and add the whites well beaten.—Flavor with lemon or vanilla.

**Meat Eggs.**

Take equal quantities of bread crumbs and finely chopped meat, a table-spoonful of flour, a little allspice, salt, one or two table-spoonsful of dripping and half an onion cut very fine. If too dry, add a little milk or good gravy; make into egg-shaped balls and fry brown in boiling lard.

These will be found very palatable, as well as an economical method for using odd bits of cold meat and bread.

**Lemon Pie.**

Two large lemons; grate the rinds and squeeze the juice. Two cups of sugar, yolks of four eggs, two large table-spoonsful of cornstarch dissolved in a cup of milk, to be added last. Line a couple of pie plates with a good pie paste, pour in the custard and bake in quick oven. Beat the whites with four table-spoonsful of pulverized sugar, until they are very light.—When the pies are done, spread this on the tops and return them to the oven until they become a very delicate brown.

**Oyster Pie.**

Strain the liquor from a quart of oysters and put it on to boil, with butter, pepper, a little salt, and thickening with a pint of bread crumbs and a cup of milk well beaten together; after boiling a few minutes, throw in the oysters.—Let them remain five minutes, take them off and when warm add the beaten yolks of three eggs. Line a buttered dish with a rich paste, fill it with white paper or a clean napkin, to support a lid of paste, and bake it. When lightly browned, take off the lid, remove the napkin, pour in the oysters, set a few minutes in the oven, and send to the table hot. Try it.

**Pound Cake.**

Three-quarters of a pound of butter, one pound of pulverized sugar, one pound and four ounces of flour, ten eggs, two table-spoonsful of brandy, and half teacup of milk, one even teaspoon of soda, two of cream of tartar.

Weigh and measure all of your ingredients. Line the bottom of the cake tin with buttered paper, mix the cream of tartar with a portion of the flour, reserve a little of the milk in which to dissolve the soda. Separate the eggs, and if you have no assistant, beat the yolks very light; you will then be ready to mix the cake. Beat the butter and sugar together until they are very light, then add alternately the yolks, flour, and milk, next the whites beaten very stiff, and lastly the brandy and soda. Beat lightly for a few minutes, turn into your tin, place in a moderate oven; when sufficiently risen, the heat may be slightly increased. Bake from one hour to an hour and a half.

COPIOUS PERSPIRATION.—A young medical student, who had been screwed very hard at his examination for admission to the faculty, on a very warm day, was nearly overcome by the numerous questions put to him, when the following query was added: "What course would you adopt to produce a copious perspiration?" After a long breath he observed, wiping his forehead, "I would have the patient examined before the Medical Society."

THE FATE OF IO.—The late lamented Lampriere tells us, says *Punch*, that Io was changed into a heifer; but we have lately gleaned from a doctor's prescription the following piece of information respecting the end of that young person:

"Io-dide of Potassium."

